# Neurotoxin-Derived Optical Probes for Biological and Medical Imaging

**DOI:** 10.1007/s11307-023-01838-1

**Published:** 2023-07-19

**Authors:** Pinar Helin Ergen, Susan Shorter, Vasilis Ntziachristos, Saak Victor Ovsepian

**Affiliations:** 1 Faculty of Engineering and Science, University of Greenwich London, Chatham Maritime, Kent, ME4 4TB United Kingdom; 2 Chair of Biological Imaging at the Central Institute for Translational Cancer Research (TranslaTUM), School of Medicine, Technical University of Munich, 81675 Munich, Germany; 3 Institute of Biological and Medical Imaging, Helmholtz Zentrum München (GmbH), 85764 Neuherberg, Germany; 4 Munich Institute of Robotics and Machine Intelligence (MIRMI), Technical University of Munich, 80992 Munich, Germany; 5 DZHK (German Centre for Cardiovascular Research), partner site Munich Heart Alliance, Munich, Germany

**Keywords:** Animal toxins, Ion channels, Fluorescent probes, ICG, Optical imaging, Visualisation, Advanced biomaterials

## Abstract

The superb specificity and potency of biological toxins targeting various ion channels and receptors are of major interest for the delivery of therapeutics to distinct cell types and subcellular compartments. Fused with reporter proteins or labelled with fluorophores and nanocomposites, animal toxins and their detoxified variants also offer expanding opportunities for visualisation of a range of molecular processes and functions in preclinical models, as well as clinical studies. This article presents state-of-the-art optical probes derived from neurotoxins targeting ion channels, with discussions of their applications in basic and translational biomedical research. It describes the design and production of probes and reviews their applications with advantages and limitations, with prospects for future improvements. Given the advances in imaging tools and expanding research areas benefiting from the use of optical probes, described here resources should assist the discovery process and facilitate high-precision interrogation and therapeutic interventions.

## Introduction

The main objective of biological imaging is uncompromised visualisation of the structure and function of living organisms in their unperturbed environments. In this pursuit, fluorescence markers and reporter proteins combined with precision delivery and spectral multiplexing have been of critical importance. Through fluorescence effects, optical imaging provides not only insights into the molecular content and structure of living systems but allows visualisation of dynamic processes in real-time, from macroscopic to subcellular and molecular levels *in vitro* and *in vivo* [1–4]. The use of fluorescence proteins and probes with specificity for various cellular and molecular interactions has greatly enhanced the imaging of functional processes with their characterisation [5–10]. These advances have been bolstered by endoscopic and hybrid capacities, which enabled capturing multiple features at unprecedented depth, with increasing precision and specificity [11–15].

Improving targeting and delivery of fluorescence probes to various types of cells and subcellular compartments has been an essential part of recent advances in optical imaging [16–21]. Fluor-labelled peptides, functionalized nanocomposites and particles are increasingly considered for a range of preclinical and clinical use, to improve the specificity and efficacy of payload delivery, and to achieve higher imaging sensitivity and contrast, with lower toxicity in single and high throughput studies [22–24]. These developments have pushed the limits of optical imaging to new realms, enabling superb visualising capabilities, from molecular and sub-molecular to system and organism levels [4, 5, 14, 25, 26]. The high specificity and potency of biological toxins owed to a major extent to targeting distinctive sets of molecules are of special interest for precision delivery [27–31]. Since their rise in early living forms, biological toxins have been continuously diversified and refined by selective pressure for higher potency, stability and specificity [32–35]. Due to the critical role of ion channels and neurotransmitter receptors in supporting essential mechanisms and functions of living organisations, they have become favourite targets of biological toxins, including those acting on peripheral and central nervous systems [27, 31, 36, 37].

**Fig. 1:**
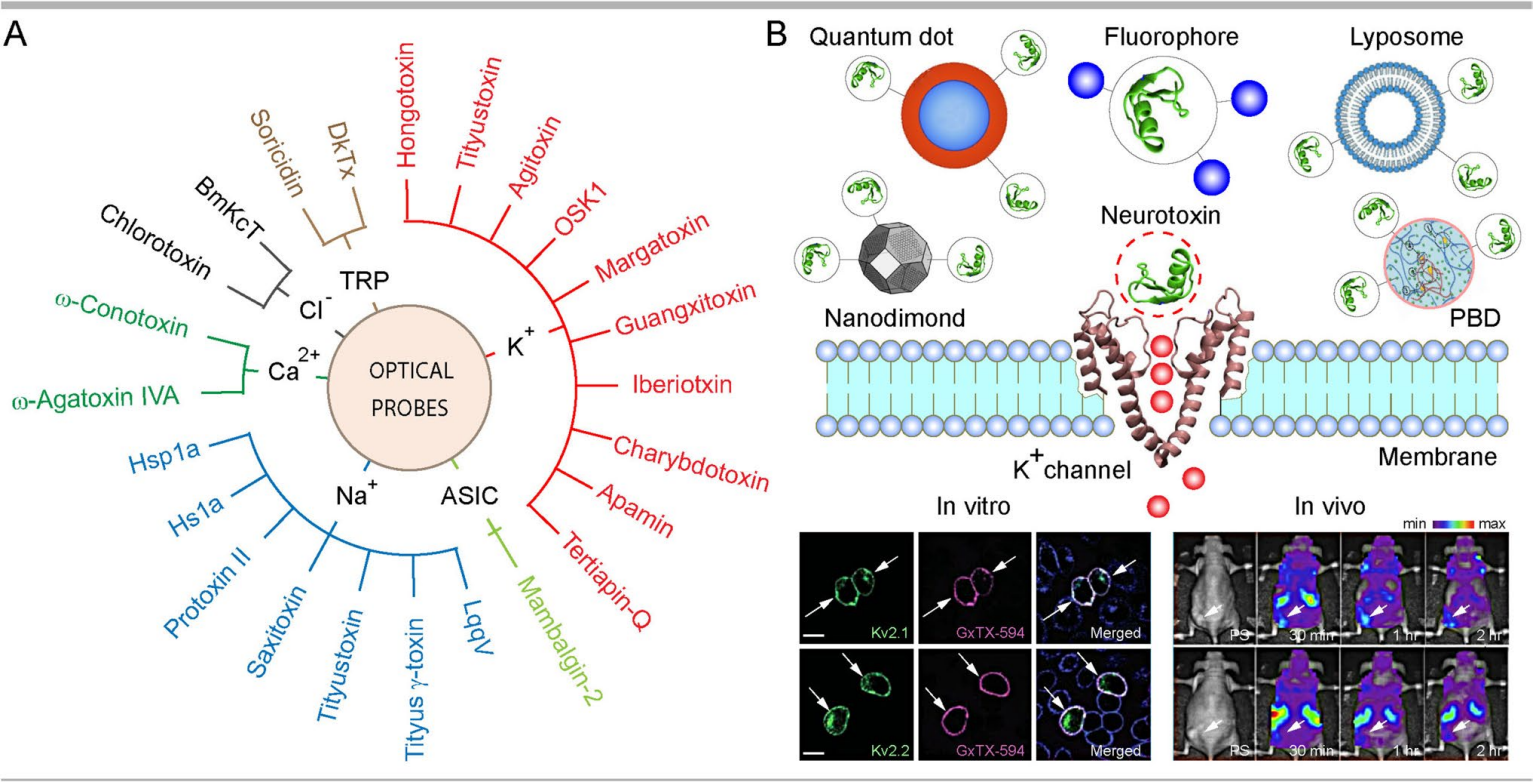
An overview of animal toxins targeting ion channels used for biomedical imaging. **A** Representation of major neurotoxins with target ion channels utilised for optical imaging in basic and translational studies. **B** Illustration of general strategy and approaches used for targeting neurotoxin-derived optical probes to ion channels with a representative *in vitro* (CHO cells expressing K_V_2.1 and K_V_2.2 labelled with GxTx labelled with Alexa594) and *in vivo* (mice grafted with SKOV3 tumours labelled with SOR-C27-Cy5.5) data. Images are reproduced with permission, with detailed explanations provided in respective parts of the review

In pursuit of improving the delivery of fluorescence probes and reporter proteins to a specific cell and tissue type, natural toxins and their detoxified variants have produced considerable interest [30, 31, 38–40]. With a growing number of recombinant forms with lower toxicity, higher specificity and delivery capacity, the expanding portfolio of biological toxins offers an extensive selection of probes and nano-carriers for a range of applications. In this article, we review the state-of-the-art optical probes derived from natural and recombinant animal toxins targeting ion channels and using them for biological and medical imaging (Fig. 1). We discuss reports exploiting neurotoxin-derived probes with specificity for potassium, sodium, calcium, chloride, TRP, acid-sensing and piezo channels, in native and heterologous systems. We conclude our analysis with considerations of key advantages and limitations, with prospects for future improvements of the use of neurotoxin-derived probes in basic and translational research.

## Optical Probes Targeting Potassium Channels

Potassium channels are one of the largest ion channel families [41, 42]. They are present in many cell types, with expression level and activity altered in a variety of diseases, including several types of cancer [43, 44], neurological and psychiatric conditions [45–48]**,** neurodegenerative diseases [49, 50] and others. Due to their extensive presence in various cell types and biological importance, K^+^ channels have been a major target of animal toxins, with many exquisitely adapted to bind and interfere with their activity and functions.

### Shaker-Related K^+^ Channels

**Fig. 2:**
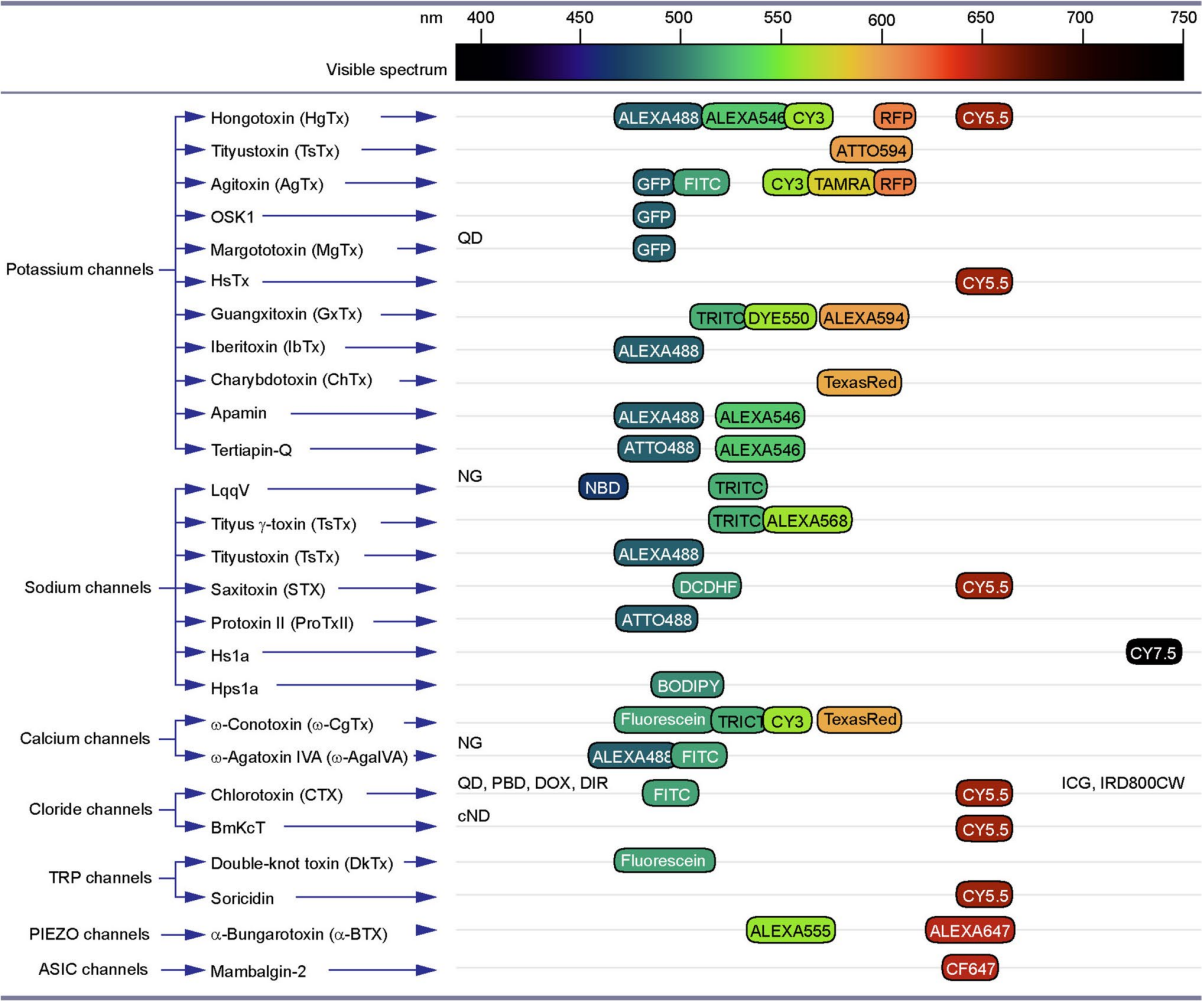
Animal toxin-derived optical probes targeting ion channels for biomedical research and clinical application. List of ion channel targets (left column) of animal toxins (middle column) used for research tagged with fluorophores, reporter proteins and nanomaterials (right column). Fluorophores and reporter proteins are aligned against the visible spectrum bar (top) to illustrate their peak absorbance. Fluorescence labels and reporters used for targeted toxins with a wide absorbance range or with a peak absorbance outside of the visible spectrum are listed on the left and right margins of the right column, respectively. QD, quantum dots; NG, nanogold; PBD, polymer-blend dots; DOX, doxorubicin; DIR, dye R; cFND, fluorescent nanodiamonds with carboxyl; ICG, indocyanine green; IRD800CW, infrared dye 800CW

Shaker channels are the most diverse subfamily of K^+^ channels, formed by the tetramerization of K_V_1.1-1.8 subunits [45, 51]. Numerous reports suggest labelled toxins targeting these channels as useful probes for visualising a range of biological processes (Table 1, Fig. 2). Hongotoxin-1 (HgTx1) of scorpion *Centruroides limbatus* and its recombinant HgTx1-A19C variant derived by site-directed mutagenesis tagged with Cy and Alexa fluorophores were developed and proved highly effective for mapping the distribution of K_V_1.1 and K_V_1.2 subunits in basket cell terminals of the rabbit cerebellum [52]. The high affinity of HgTx-A19C to the K_V_1.3 subunit rendered Cy5-HgTx-A19C also useful for 3D imaging of K_V_1.3 expression and distribution in Jurkat cells at a single molecule level [53]. The location of channels has been resolved ~40 nm along the x and y axis, with their distribution and dynamics across the cell membrane described. Selective labelling of K_V_1.3 in Jurkat cells with Cy5-HgTx-A19C and imaging of clustered channels have been confirmed by another report [54]. Using ATTO594-labelled Tityus toxin (TsTx) derived from scorpion *Tityus serrulatus*, it was possible to visualise K_V_1.2 subunits of the molecular layer and Purkinje cells of the rat cerebellum, including the pinceau regions of basket cells, confirming TsTx targeting the basket cell axon terminals and Purkinje cell dendrites [55]. Finally, the selectivity of scorpion *Leiurus quinquestriatus hebraeus* derived agitoxin-2 (AgTx-2) for K_V_1.3 subunit enabled visualisation of channels containing this protein in transfected HEK293 cells, using TAMRA labelling (AgTx-2-D19C-TAMRA). Through confocal imaging, the surface expression of K_V_1.3 in HEK293 cells was confirmed (https://www.alomone.com/p/agitoxin-2-cys-tamra/RTA-420-T). Another study used AgTx-2 labelled with FITC and Cy3 to elucidate the relationship of K_V_2.1 and K_V_10.2 with actin filaments, as well as the distribution and clustering of K_V_1.1, K_V_2.1 and K_V_10.2 in transfected green monkey kidney Vero cells [56]. Whilst there was no association of actin filaments stained with Alexa488-phalloidin with K_V_2.1 channels, a strong co-localization of actin was observed with K_V_10.2, supporting differential sorting and trafficking of these channels through interactions with actin filaments [56]. The utility of quantum dot (QD)–tagged toxins for imaging K_V_1.3 has been also shown using margatoxin (MgTx) obtained from the venom of the scorpion *Centruroides margaritatus* [57, 66]. MgTx linked via amino group-carboxylic crosslinker with QD CdSe-ZnS core-shell nanocrystals showed high selectivity to K_V_1.3 [57]. To demonstrate the specificity and high quantum efficacy of QDs-MgTx, it was applied to HEK293 cells transfected with GFP tagged K_V_1.3 construct, and showed a strong co-localisation of QDs with GFP, yielding a more stable and stronger QD signal [57]. A very recent study showed that conjugation of the scorpion *Heterometrus spinifer*-derived HsTX1 analogue HsTX1 [R14A] with Cy5 allowed the visualisation of GFP-K_V_1.3 channels in CHO cells [58]. Cy5-HsTX1 enabled also imaging of Kv1.3 in BV-2 microglia cells of C57BL/6 mice treated with lipopolysaccharides (LPS), known to upregulate K_V_1.3 subunit. The presence of fluorescence signals in the kidney, intestine and liver of C57BL/6 mice injected with Cy5-HsTX1 [R14A] implies its potential usefulness for biodistribution studies. Authors suggest that Cy5-HsTX1 [R14A] is a useful probe to determine the location and distribution of K_V_1.3 channels under physiological as well as autoimmune and neuroinflammatory conditions, associated with the upregulation of K_V_1.3 channels [58].

**Table 1:** Optical probes targeting potassium channels

Target, ion channel	Ligand (toxin)	Fluor-reporter	Model	Ref.
K_V_1.1, K_V_1.2	HgTx1	Cy3, Alexa488, 546	Cerebellum, rabbit	[52]
K_V_1.3	HgTx1	Cy5	Jurkat cell line	[53]
K_V_1.3	HgTx1	Cy5	Jurkat cell line	[54]
K_V_1.2	TsTx	ATTO594	Cerebellum, rat	[55]
K_V_2.1, K_V_10.2	AgTx-2	FITC	Vero cells	[56]
K_V_1.1	AgTx-2	Cy3	Vero cells	[56]
K_V_1.3	MgTx	Quantum dots	HEK293 cells	[57]
Kv1.3	HsTx1	Cy5	CHO and BV-2 cells, mouse	[58]
K_V_1.3	AgTx-2	TAMRA	HEK293 cells	AL
K_V_1.1, KcsA-K_V_1.1	HgTx1	ATTO488	*E. coli*	[59]
K_V_1.3, KcsA- K_V_1.3	AgTx-2	TAMRA	*E. coli*	[60]
KcsA-K_V_1,x (x;1,3,6)	AgTx-2	Tag-RFP	*E. coli*	[61]
KcsA-K_V_1.x (x;1,3,6)	OSK1	eGFP	*E. coli*	[61]
K_V_1.3, KcsA- K_V_1.3	AgTx-2	GFP	*E. coli*	[62]
K_V_1.1, KcsA-K_V_1.1	HgTx1	Tag-RFP	*E. coli*	[63]
K_V_1.1, KcsA- K_V_1.1, KcsA- K_V_1.3	HgTx1	Tag-RFP	*E. coli*	[64]
K_V_1.3, KcsA- K_V_1.3	MgTx	GFP	*E. coli*	[65]
K_V_2.1	GxTx	Dye550	CHO-K1 cells	[70]
K_V_2.1	GxTx	TMR	Hippocampus, rat	[70]
K_V_2.1	GxTx	Alexa594	CHO cells, hippocampus, rat	[71]
BKCa	IbTx	Alexa488	HEK293 cells	[72]
BKCa	IbTx	Alexa488	Cochlear hair cells, mouse	[73]
BKCa	ChTx	Texas red	Neuromusc. junction, frog	[74]
SKCa	Apamin	Alexa488, 546	Hippocampal, rat	[75]
SKCa	Apamin	Alexa488, 546	Hippocampus, rat	[76]
Kir3.1, Kir3.4	Tertiapin-Q	ATTO488	HEK293 cells	[77]
Kir1.1	Tertiapin-Q	FTIC-AuNP	PC12 cells	[78]

### Recombinant K_V_ Channels with Shaker Channel Binding Domains

In addition to visualising native channels, venom toxins were used also for imaging chimeric K^+^ channel proteins (KcsA-K_V_1,x; x=1,3,6) made by transfer of the eukaryotic (K_V_) S5-S6 linker region within the pore domain in bacterial homologues (KcsA) [67]. The specific binding of ATTO488-labelled HgTx [59] and TAMRA-tagged AgTx-2 [60] with KcsA-K_V_1.1 and KcsA-K_V_1.3 chimaeras expressed in *E. coli* spheroplasts enabled their visualisation using confocal imaging. Likewise, in *E. coli* with and without recombinant KcsA-K_V_1.x (1,3,6), OSK1-eGFP (toxin derived from scorpion *Orthochirus scrobiculosus*) and AgTx-2-TagRFP were reported to be effective for selective labelling K_V_1 channels [61]. Notably, AgTx-2 with GFP at its N-terminus exhibits high specificity for chimeric K_V_1.3 channels over K_V_1.1 and K_V_1.6 in spheroplasts, and KcsA-K_V_1.3 transfected HEK293 cells [62]. Similar studies with the fusion of HgTx with Tag-RFP showed maintained specificity and utility for targeting KcsA-K_V_1.1 [63] as well as both KcsA-K_V_1.1 and KcsA-K_V_1.3 [64]. Finally, GFP-MgTx fusion protein revealed specific labelling of the K_V_1.3 subunits of the KcsA-K_V_1.3 hybrid channels expressed in *E. coli* spheroplasts, whilst control experiments with non-transfected material showed no fluorescence signal [65].

### Shab K^+^ Channels

Two members of this family, K_V_2.1 and K_V_2.2 multimerize to form functional channels mediating delayed rectifier currents [68, 69]. Genetically modified (S13C) and fluor-labelled guangxitoxin (GxTX) variants from tarantula *Plesiophrictus guangxiensis* venom were used to study K_V_2.1 channels of transfected CHO cells and visualise endogenous K_V_2.1 channels of rat hippocampal pyramidal neurons [70, 71]. Tetramethylrhodamine (TMR, known also as TRITC) and Dye550-labelled GxTx enabled imaging of K_V_2.1 channel localization and expression, with binding activity depending on voltage changes in CHO-K1 cells [71], whilst Alexa Fluor 594-GxTx has been used to visualise the expression of K_V_2.1 in transfected CHO cells, demonstrating a correlation of fluorescence signal with membrane voltage. Also, Alexa594-GxTx enabled surface labelling of K_V_2.1-GFP transfected rat CA1 pyramidal neurons [70].

### Ca^2+^-Activated K^+^ Channels

For mapping of big conductance calcium-activated potassium channels (BK_Ca_) on the surface of living HEK293 cells, the venom of scorpion *Mesobuthus tamulus* iberiotoxin (IbTx) has been chemically modified and linked with Alexa488. Alexa488-IbTx showed a strong signal on the membrane surface of BK_Ca_-expressing HEK293 cells, whereas no fluorescence was observed in non-transfected HEK293 cells [72]. Application of a recombinant iberiotoxin (IbTx-D19C) tagged with Alexa488 in mouse inner hair cells revealed the strong expression of BK_Ca_ [73]. Earlier work with the use of a similar approach showed the utility of the Streptavidin-Texas Red-labelled biotinylated-charybdotoxin (ChTx) derived from the venom of scorpion* Leiurus quinquestriatus hebraeus* as an imaging probe for BK_Ca_ channels, which were enriched in proximity to presynaptic Ca^2+^ channels at synaptic terminals of frog neuromuscular junctions (NMJ) [74]. Abiraman and co-workers used Alexa488- and Alexa546-streptavidin-tagged apamin isolated from the bee venom to map the distributions of Small Conductance K_Ca_ channels (SK_Ca_^+2^) in somatodendritic compartments of hippocampal neurons in culture [75]. Imaging of channels with fluor-labelled biotinylated-apamin confirmed the hypothesis that SK channels of hippocampal neurons are enriched in the initial segment of axons [76].

### Inward Rectifier K^+^ Channels

Isolated from honeybee venom, tertiapin-Q shows high selectivity for inward rectifier K^+^ (Kir) channels. Fluor-labelled tertiapin-Q-ATTO488 was used as a tool to visualise the localization and monitor the dynamics of Kir3.1 and Kir3.4 in HEK293 cells in a study of the effects of small molecule (drug chloroquine) blocker of Kir channels in atrial fibrillation [77]. Another example of the successful use of fluor-labelled tertiapin-Q is FITC fluorescein-labelled gold nanoparticle conjugated with tertiapin-Q (TPN-Q-AuNP/FITC), which helped to visualise the Kir1.1 channel upregulation in pheochromocytoma cell (PC12) by nerve growth factor (NGF) [78]. Results of imaging studies confirmed the specificity of TPN-Q-AuNP to various Kir channels in PC12 cells, with the potential utility for a wide range of research applications.

## Optical Probes Targeting Sodium Channels

The sodium channel family is the second most diverse group of ion channels, playing a key role in membrane excitability, and the generation and propagation of action potentials in neurons, muscle cells and other excitable tissue [79, 80]. Because of the critical importance of Na^+^ channels in cellular activity, they have been a favourite target of many biological toxins [81, 82], which present significant interest as probes for selective labelling of various cell types and their functional processes, as well as for delivery of therapeutic candidates (Table 2, Fig. 2).

**Table 2:** Optical probes targeting sodium channels

Target, ion channel	Ligand (toxin)	Fluor-reporter	Model	Ref.
Na_V_	LqqV	DNB	Sciatic nerve, mouse	[87]
Na_V_	LqqV	TmRhd	Myotubes, chick	[88]
Na_V_	LqqV	TmRhd	Spinal cord, rat	[89]
Na_V_	TiTx- γ	TmRhd	Cortex, rat	[89]
Na_V_	TiTx- γ	TmRhd	Dorsal root ganglion, rat	[90]
Na_V_	TiTx- γ	Alexa568	GH3 cells	[91]
Na_V_	TsTx	Alexa488	GH3 cells	[91]
Na_V_	STX	Cy5	PC12 cells	[92]
Na_V_	STX	DCDHF	PC12 cells	[92]
Na_V_1.7	ProTx II	ATTO488	CHO cells	[93]
Na_V_1.7	Hsp1a	BODIPY	Sciatic nerve, mouse	[94]
Na_V_1.7	Hs1a	Cy7.5	HEK293 cells	[95]

### Pan-Na Channels

Animal toxins have been critical in the characterisation of the structure and function of Na^+^ channels [83, 84], with tetrodotoxin (TTX) and saxitoxin (STX) playing an important role in describing the channel properties, structure of the selective filter and toxin binding [85, 86]. A wide variety of animal toxins targeting Na^+^ channels have been described, with some successfully used for imaging. Angelides and Nutter were the first to use fluorescence tetrodotoxin of pufferfish *Takifugu obscurus*, and 2,6-dimethylnitrobenzene (DNB) conjugated LqqV toxin of scorpion *Leiurus quinquestriatus* venom to target Na^+^ channels, mapping their expression in nodal regions of myelinated fibres of mouse sciatic nerves, enhanced by immunohistochemical staining [87]. A follow-up report with tetramethylrhodamine dextran (TmRhd)-LqqV showed enrichment of Na^+^ channels at the NMJ of embryonic chick myotubes with co-cultured spinal cord neurons [88]. To identify regions where Na^+^ channels are densely expressed, neurons were treated with neurotoxin conjugates NBD-LqqV, TmRhd-LqqV, CPM-Css II, NBD-TTX and TmRhd-Tityus-γ [89]. It was found that in cortical neurons, Na^+^ channels are enriched in the neuronal soma and reach the highest density in the axon hillock, in agreement with the latter as the site of initiation of action potentials. Another study investigated the mobility of sodium channels during myelination, using TmRhd-Tityus-γ application to cultured dorsal root ganglion neurons in the presence and absence of Schwann cells (SCs), which change the distribution of Na^+^ channels in axons from diffuse to clustered in the nodal region [90]. Fluorescence photobleaching recovery measurements in this model showed that SCs did not affect sodium channel lateral mobility in the membrane.

In addition to labelling neurons, TsTx (α-type) and TiTX-γ (β-type) of scorpion *Tityus serrulatus* venom conjugated with Alexa488 and Alexa568 fluorophores, respectively, were used to target Na_V_ channels in living GH3 cells, to visualise their distribution [91]. The specificity of labelling was proven by the preincubation of cells with naive toxins or treatment of non-transfected HEK293 cells with the labelled toxin. Like in *Tityus serrulatus* toxins studies, STX of paralytic shellfish poison labelled with N-hydroxysuccinimde (NHS) derivatives of Cy5 and DCDHF fluorophores enabled mapping of Na_V_ distributions in NGF differentiated PC12 cells, with antibody labelling of Na^+^ channels verifying the staining specificity [92]. Finally, STX-Cy5 allowed the visualisation of channel distributions in living cells at the single molecule level, whilst the use of super-resolution methods made possible STX-Cy5 imaging of neuritic spines and filopodia in NGF-differentiated PC12 cells [92].

### Na_V_1.7 Channels

To visualise surface expression and distribution of Na_V_1.7 channels in neurons, fluorescence labelling of Protoxin II (ProTx II) from the venom of tarantula spider *Thrixopelma pruriens* with selectivity to Na_V_1.7 was used [93]. ATTO488-tagged ProTxII was applied on CHO cells expressing human Na_V_1.7, on naïve CHO cells lacking Na_V_1.7 channels, and on dorsal root ganglion (DRG) neurons expressing endogenous Na_V_1.7. These experiments resulted in the labelling of Na_V_1.7 channel expressing CHO cells, as well as the proximal neurite parts and soma of DRG neurons [93]. The inability of ATTO488-ProTxII to bind labeled CHO cells pre-incubated with non-labelled ProTxII proved the toxin specificity for Na_V_1.7 channels. Another report used BODIPY-FL-NHS ester dye-conjugated Hsp1a peptide toxin from the venom of the tarantula *Homoeomma spec,* known for its selectivity for Na_V_1.7 channels [94]. Injection of this probe in mice resulted in high-intensity fluorescence signals of sciatic nerves tested *in vivo* and *ex vivo* [94]. Similarly, labelling a recombinant peptide analogue of Hs1a isolated from the venom of the Chinese bird spider *Haplopelma schmidti* with the Cy7.5 made visible Na_V_1.7 expressed in mouse sciatic nerves and enabled their near-infrared imaging in nodes of Ranvier [95].

## Optical Probes Targeting Calcium Channels

Calcium channels are a family of proteins forming Ca^2+^ selective pores, which play a key role in a wide range of biochemical and electrophysiological processes in neurons and other cells, including control of molecular signalling, regulation of secretory processes, excitability and action potentials, gene regulation and others [96, 97]. Like other prevalent ion channels, Ca^+2^ channels have been a major target for a range of biological toxins [36, 98]. Due to the high affinity and selectivity of some of the toxins for Ca^+2^ channels, their labelling has been utilized for studies of channel distribution and function *in situ* and heterologous expression models (Table 3, Fig. 2).

**Table 3:** Optical probes targeting calcium channels

Target, ion channel	Ligand (toxin)	Fluor-reporter	Model	Ref.
P/Q-type	ω-Aga IVA	FTIC	Hippocampus and cerebellum, mouse	[102]
P/Q-type	ω-Aga IVA	Alexa488-nanogold	Brainstem, mouse	[103]
N-type	ω-CgTx	TmRdh	Hippocampus, rat	[104]
N-type	ω-CgTx	TexasRed	Neuromuscular junction, frog	[105]
N-type	ω-CgTx	TmRhd	Motor nerve, frog	[106]
N-type	ω-CgTx	TmRhd	Cerebellum, mouse	[107]
N-type	ω-CgTx	Fluorescein	Hippocampus, rat	[108]
N-type	ω-CgTx	Fluorescein	Hippocampus, rat	[109]
N-type	ω-CgTx	Cy3	Hippocampus, rat	[110]

### P/Q Type Channels

Derived from the funnel-web spider *Agelenopsis apera* venom, ω-agatoxin IVA (ω-Aga IVA) has superb affinity and selectivity for P/Q type voltage-gated Ca^2+^ channels [99–101]. Using confocal microscopy of mouse brain tissue, biotinylated ω-Aga IVA labelled with FITC-avidin D enabled mapping of the distribution of P/Q Ca^2+^ channels in the mouse brain, particularly in the cerebellum and hippocampal region [102]. Based on the imaging results, it was concluded that P/Q Ca^2+^ channels are expressed in soma and dendrites of Purkinje cells, granule cells and interneurons in the cerebellum, as well as pyramidal cells of the CA1 and CA4 regions of the hippocampus. These results agree with electrophysiological data on P/Q-type Ca^2+^ channels in described neuron types and brain regions. Biotinylated ω-Aga IVA tagged with Alexa Fluor488-streptavidin nanogold (NG) was also applied to mouse auditory brainstem slices, demonstrating distinctive labelling of binding sites [103]. High-power microscopic analysis allowed visualisation of the distribution of P/Q channels, with no labelling observed in slices treated with Alexa Fluor488-streptavidin nanogold alone in P/Q channel knockout mice, confirming the selectivity of the probe [103].

### N-Type Channels

Like ω-Aga IVA, labelled ω-conotoxin (ω-CgTx) of the venom of Pacific cone snail *Conus geographus* has been used for describing the distribution of N-type Ca^2+^ channels in cells. In hippocampal CA1 neurons of rats, the application of ω-CgTx labelled with a colloidal gold particle and TmRdh enabled visualisation of N-type Ca^2+^ channels of the soma and dendrites of neurons with clustering on synaptic terminals [104]. The feasibility of imaging of Ca^2+^ channels in nerve terminals of frog NMJ and their interactions with acetylcholine receptors was shown using ω-CgTx conjugated with Texas red [105]. It was found that Ca^2+^ channels at NMJ colocalize with acetylcholine receptors, verified also by α-bungarotoxin staining. Similarly, conjugation of ω-CgTx with the succinimidyl ester of TmRhD strongly labelled Ca^2+^ channels clustered at active zones of presynaptic terminals in frog motor nerves [106].

Microscopic analysis with TmRhd-ω-CgTx revealed the presence of N-type Ca^2+^ channels on the surface of postmitotic granule cells in developing mouse cerebellar slices, with their expression maintained in migrating and maturating neurons [107]. Studies of living rat hippocampal brain slices labelled with fluorescein-ω-CgTx conjugate allowed visualisation of N-type Ca^2+^ channels in all hippocampal regions, along with their distribution in various neuronal compartments and dendritic spines [108]. The distribution of N-type Ca^2+^ channels was also analysed during the development of rat brains [109]. Finally, the same approach using ω-CgTx-Cy3 allowed the detection of the changes in the expression of N-type Ca^2+^ channels in hippocampal slices of the kindling model of rat epilepsy [110]. Analysis of the labelling of dendrites of CA1 and CA3 neurons in epilepsy rat models with CgTx-Cy3 showed alterations in signal intensity compared to controls, suggesting changes in the prevalence of N-type Ca^2+^ channel in epilepsy disorders.

## Optical Probes Targeting Chloride Channels

Chloride channels are a functionally and structurally diverse group of anion-selective proteins involved in the regulation of a range of functions in cells, including membrane potential, cell volume and cycle and apoptosis [111, 112]. Cl^-^ channels are classified into several subfamilies [113], which are differentially expressed in various cell types under physiological and disease conditions [114, 115]. Like K^+^ and other cation channels, specific enrichment of some Cl^-^ channels has been observed in neoplastic tissue, which renders them useful for molecular imaging and targeting therapies.

### Small Conductance Chloride Ion Channels

Chlorotoxin (CTX) of the ﻿deathstalker scorpion *Leiurus quinquestriatus* [116, 117] is a selective ligand for Cl^-^ channels, also known to bind matrix metalloproteinase-2 (MMP-2) and annexin-2 on the cell membrane [118–120] (Table 4, Fig. 2). Cy5.5 conjugation with NHS ester to CTX allowed imaging of the 9L rat glioma cells *in vitro* and glioma xenograft mice, as well as in brain slices of ND2:SmoA1 medulloblastoma model [121]. The specificity of labelling was verified using a competitive assay with nonlabelled CTX. CTX-Cy5.5 also enabled imaging of prostate cancer in epithelium and lymph nodes, lung metastases and sarcoma in mouse cancer models. Substitution of lysine 15 and lysine 23 residues of CTX (K15A K23ACTX:Cy5.5 or K15R K23RCTX:Cy5.5) allowed prevention of mono-, di-, tri-labelling, which causes complications with quantitative imaging, and enables mono- lysine 27 labelling [122]. In the same report, cyclized CTX has been developed and used to optimise the serum stability of CTX bioconjugates [122]. It has been found that substituted and cyclized Cy5.5 labelled CTX retained the specificity and displayed the ability to selectively label tumour regions in ND2:SmoA1 medulloblastoma models [122].

**Table 4:** Optical probes targeting chloride channels

Target, ion channel	Ligand (toxin)	Fluor-reporter	Model	Ref.
MMP-2 (Cl^-^)	CTX	Cy5.5	9L cells (in vitro); glioma, prostate, lung metastases xenograft mice, ND2SmoA1 medulloblastoma mice	[121]
MMP-2 (Cl^-^)	CTX	Cy5.5	ND2:SmoA1 medulloblastoma mice	[122]
MMP-2 (Cl^-^)	CTX	ICG	LN229 xenograft mice	[123]
MMP-2 (Cl^-^)	CTX	ICG	Dog models with various solid tumours	[125]
MMP-2 (Cl^-^)	CTX	ICG	Head and neck squamous carcinoma	[126]
MMP-2 (Cl^-^)	CTX	ICG	Human skin cancer lesions	[127]
MMP-2 (Cl^-^)	CTX	IRD-800CW	U87MG xenograft mice, NS2:SmoA1 mice	[128]
MMP-2 (Cl^-^)	CTX	PEG-iron oxide-Cy5.5	ND2:SmoA1 medulloblastoma mice	[129]
MMP-2 (Cl^-^)	CTX	Magnetic iron oxide-FTIC	U251MG, C6 cells	[130]
MMP-2 (Cl^-^)	CTX	NaYF(4):Yb,Er/Ce	C6 cells, C6 xenograft mice	[131]
MMP-2 (Cl^-^)	CTX	DOX-liposomes	U251MG, U87 and C6 cells	[132]
MMP-2 (Cl^-^)	CTX	Dir-liposomes	U87, xenograft mouse	[132]
MMP-2 (Cl^-^)	CTX	DOX-liposomes	4T1 cells	[133]
MMP-2 (Cl^-^)	CTX	Dir-liposomes	4T1, xenograft mice	[133]
MMP-2 (Cl^-^)	CTX	AIS/Zns QD/PLGA-PEG MI	U87 cells	[134]
MMP-2 (Cl^-^)	CTX	C-6 loaded PLGA, nano	GI-1, U87 cells	[135]
MMP-2 (Cl^-^)	CTX	Nano-agent, ICG	U87MG, xenograft mouse	[136]
MMP-2 (Cl^-^)	CTX	Polymer-blend dot	ND2:SmoA1 medulloblastoma mice	[137]
MMP-2 GCC	BmKcT	Cy5.5	C6 cells	[142]
MMP-2 GCC	BmKcT	Carboxylated-ND	C6 cells	[143]

The utility of peptide fluorophore CTX-indocyanine green (ICG), known also as BLZ-100, for surgical guidance and resection of malignant tissue was reported by numerous studies [123, 124]. BLZ-100 accumulated in the neoplastic area of LN229 human glioblastoma grafted mice, allowing NIR imaging, whereas ICG alone did not produce specific labelling of the pathological tissue [123]. The ability of BLZ-100 to differentiate healthy and malignant tissue was tested also in dog models of tumours [125]. Baik and co-workers used BLZ-100 for imaging mouse xenografts of head and neck squamous cell carcinoma (HCSCC) and hamster xenografts of high- and low-risk oral dysplasia [126]. In PCI-15B xenografts expressing GFP, uptake of free ICG alone did not produce fluorescent signals, confirming the tumour-specificity of BLZ-100. Recently, human clinical trials of BLZ-100 were performed in patients with skin cancer, demonstrating specific and concentration-dependent labelling of neoplastic tissue [127]. In neuroimaging applications, CTX conjugated with infrared dye 800 CW (IRD-800CW) labelled the U87MG glioblastoma xenografts in mice, strongly colocalizing with MMP-2 expression [128]. In the same study, brain slices of ND2:SmoA1 genetically modified medulloblastoma mice treated with IRD-800CW-CTX exhibited a higher fluorescence than in tumour-free mice [128]. Using PEG-iron oxide-CTX-Cy5.5 nanoprobes as NIR optical imaging tool, Veiseh and co-workers visualised medulloblastoma in ND2:SmoA1 model mice, with the utility for intraoperative tumour resection verified through histological analysis [129].

Meng and co-workers showed that CTX functionalized iron oxide nanoparticles linked with FITC allow the labelling of U251MG and C6 rat glioma cells [130]. Another example of functionalizing nanoparticles with CTX for imaging was the use of hexagonal-phase NaYF(4):Yb,Er/Ce (up-conversion, UCNP) labelling of root C6 rat glioma cells, which showed that in addition to enhanced accumulation of CTX-UCNP nanoprobe, the signal was specifically localised to the tumour region of C6 glioma-bearing xenograft mice [131]. Successful use of CTX-targeted liposomes (LP) has been also shown for the delivery of fluorescence load to glioma cells, allowing their visualisation with optical imaging [132]. In U251MG, U87 human and C6 rat glioma cell lines, doxorubicin (DOX)-loaded CTX-LP emitted a stronger fluorescence signal compared to non-targeted DOX-loaded LP. Unlike free DIR and blank LP, targeted DIR-loaded LP showed enhanced accumulation in U87 cell-bearing armpit and orthopaedic mouse tumour models, including in brains [132]. DOX-LP functionalised with CTX (DOX-SSL-CTX) was also utilized as an imaging probe in 4T1 murine breast cancer cells, to evaluate the uptake of liposomes [133]. As a result, higher fluorescence intensity was observed with DOX-SSL-CTX compared with CTX-free DOX-SSL. Finally, modification of Dir-loaded liposomes with CTX (Dir-SSL-CTX) was used to investigate the targeting efficiency of liposomes in 4T1 cells bearing BALB/c mice *in vitro*. Authors conclude that CTX enhances the LP targeting and cargo internalisation in metastatic breast tumours [133].

CTX has been also successfully utilised for functionalizing AIS/ZnS quantum dots (QD) with Polylactide-co-glycolide (PLGA)-polyethylene glycol (PEG) micelles to U87 brain tumour cells [134]. Pre-treatment of the U87 cells with MMP-2 inhibitor before AIS/ZnS QDs treatment resulted in a decrease in fluorescence signal. These results demonstrated the effectiveness of CTX as a tool for targeted imaging of biological specimens using AIS/ZnS QDs. CTX has also been shown to enhance the targeting of PLGA nanoparticles loaded with anti-cancer mourisin (MOR) to glioma cells [135]. Nanoparticles loaded with coumarin-6 (C-6) conjugated to CTX (instead of MOR) and free nanoparticles were applied in HCN-1A, GI-1 and U87 cells in culture. Results of these studies show that whilst human neuronal cell line HCN-1A displayed a weak fluorescence signal due to lack of MMP-2 receptor, the fluorescence signal was stronger in glioma GI-1 and U87 cell lines. Patil and co-workers developed polymalic acid (PLMA) nanoparticle platform with tri-leucine peptide (LLL) and ICG, with CTX used for their targeting [136]. Selective labelling of U87MG glioma-bearing xenograft by PLMA-LLL-ICG-CTX was observed in a mouse brain, but not in tumour-free tissue. CTX and tri-leucine peptide-free PLGA-ICG showed little or no signal, whilst tri-leucine peptide-free agents showed a lower signal than PLMA-LLL-ICG-CTX [136]. Finally, Wu and co-workers applied CTX targeting to polymer-blend dots (Pbdot, PBD), which are ~15 times brighter than QDs at 655nm and show significantly higher stability in serum, with no toxicity. Authors report strong and specific Pdot-CTX ND2:SmoA1 labelling of medulloblastoma of mouse brain *ex vivo*. Contrary to this, there was no signal in ND2:SmoA1 with control Pbdot-PEG and in C57BL/6 wild-type mice lacking brain tumours [137].

### Glia-Specific Cl^-^ Channel

There have been reports of the expression of a specific Cl^-^ channel (GCC) in human astrocytoma cells, which could be blocked by CTX [138, 139]. Some studies have shown that chlorotoxin-like toxin BmKcT derived from the cDNA of the Chinese scorpion *Mesobuthus martensii Karsch's* salivary glands bind specifically to this channel [140, 141]. Like CTX, Cy5.5 conjugated BmKcT was tested in a glioma rat model produced by grafting C6 cells [142] (Fig. 2). BmKcT targeting caused a much stronger labelling of tumour tissue compared to non-targeted Cy5.5 [142]. Another report tested BmKCT functionalized fluorescent nanodiamonds with carboxyl (cFND) in C6 cells, and showed stronger labelling of the malignant tissue, compared to non-targeted cFND [143].

## Optical Probes Targeting TRP Channels

Over 30 transient receptor potential (TRP) channel homologues have been identified in mammals, which are divided into six subfamilies [144]. One of these, TRPV consists of six members: TRPV1-TRPV6. TRPV1-TRPV4 are enriched in the sensory system, playing a key role in detecting various stimuli such as heat, stretch, acidity and pain, and have low selectivity for Ca^2+^, whilst TRPV5-6 are highly selective for Ca^2+^ and regulate molecular signalling and housekeeping functions in a wide variety of cells [145–150].

### TRPV1 Channels

To visualise the expression and distribution of TRPV1 channels, double-knot-toxin (DkTx) from tarantula *Ornithoctonus huwena* venom [151] conjugated with fluorescein via sortase at the C-terminus was applied to *Xenopus leaves* oocytes transfected with rat TRPV1 channels [152] (Table 5; Fig. 2). Because DkTx contains a large number of cysteine residues, cysteine-mediated bioconjugation caused a high level of misfolding of the toxin. Nevertheless, the authors report that their approach enabled successful and selective labelling of rat TRPV1 in the oocyte model [152].

**Table 5:** Optical probes targeting TRPV1, PIEZO1 and ASIC1a channels

Target, ion channel	Ligand (toxin)	Fluor-reporter	Model	Ref.
TRPV1	DkTx	Fluorescein	Xenopus laevis oocytes	[152]
TRPV6	Soricidin	Cy5.5	SKOV-3 xenografts, mouse	[157]
Piezo 1	α-BTX	Alexa647	HEK293T	[158]
Piezo 1	α-BTX	Alexa555	Neuro2A	[159]
ASIC1a	Mambalgin-2	CF647	Mel P cells	[162]

### TRPV6 Channels

TRPV6 has been implicated in neoplasia, with its level enhanced in the ovary, breast, colon, prostate and thyroid cancers [153, 154]. In prostate cancer, TRPV6 mRNA levels are positively correlated to tumour progression and aggressiveness, pathological stages and extra-prostatic metastases, with poor prognosis [155, 156]. Bowen and co-workers used soricidin (accession number P0C2P6)—a paralytic peptide isolated from the submaxillary saliva glands of the northern short-tailed shrew *Blarina brevicauda*, known to inhibit Ca^2+^ influx *via* TRPV6 channels [157]. Two C-terminus peptide sequences of soricidin (SOR-C13 and SOR-C27) were shown to bind specifically TRPV6 in ovarian cancer cells [157] as well as ovarian cancer xenografts in mouse models. Conjugation of C-terminus SOR-27 peptide sequence of soricidin with Cy5.5 and superparamagnetic iron oxide, when applied *in vivo*, accumulated in the ovarian tumour regions of xenograft mice and could be visualised with fluorescence imaging [157]. Authors conclude that SOR peptides may be useful for detecting tumours and delivering diagnostic or therapeutic payloads, via targeting TRPV6 channels.

## Optical Probes Targeting Other Ion Channels

In addition to demonstrating the successful use of labelled toxins derived from animal venom for visualisation of main cation and anion-selective channels, some of the toxins were recently also successfully utilised as probes for imaging acid-sensing ion channels (ASIC) and Piezo receptor channels [158–160] (Table 5, Fig. 2).

### Piezo1 Channel

To visualise the distribution and activity of mechanoreceptive channels, Lee and co-workers tailored recombinant Piezo1 protein containing α-bungarotoxin (α-BTX) binding site, which was visualised by α-BTX conjugated with Alexa555 (BTX-Alexa555) [159]. Exposure of N2A cells transfected with Piezo1-13 residue BTX binding site to Alexa555-BTX revealed strong and specific labelling. The construct has been used for addressing functional questions related to trafficking and regulation of Piezo1/2 channels [159]. Another study used α-BTX-Alexa647 for visualising HEK293 cells transfected with Piezo1 channels containing α-BTX binding sites, to determine the mechanically sensitive domains of Piezo1 channels and their response to magnetic nanoparticles [160].

### ASIC1a Channel

A recent report by Bychkov and co-workers used modified mambalgin-2 for imaging the expression and distribution of ASIC1a channels [158]. Derived from black mamba *Dendroaspis polylepis* venom, mambalgin-2 specifically and reversibly blocks the pathological upregulation of ASIC1a in melanoma cancer associated with acidification of tumour tissue [161]. When applied on metastatic skin melanoma mel P cells, mambalgin-2 with Leu32Ala mutation labelled with the CF647 dye revealed a strong presence of ASIC1a subunit as well as colocalization with epithelial Na^+^ channel family members (α-ENaC and γ-ENaC), an observation verified with immunofluorescence microscopy [162]. Using a mutant variant of mambalgin-2 with reduced binding activity for ASIC1a, it was confirmed that the principal molecular target of mambalgin-2 in melanoma cells is the ASIC1a subunit [158].

## Summary and Future Directions

Over millennia, severe pain, intoxication and death were what drew our respect and curiosity to venomous animals. The lethal power of animal toxins demanded not only reverence, but more recently, also systematic research and understanding, which with scientific progress and technological advances facilitated the harnessing of their ability to treat an expanding range of conditions and diseases. The plethora of biologically active compounds produced by venomous animals capable of targeting receptors and ion channels, as shown here, are not only relevant to pharmacology and toxicology but also molecular imaging, offering means for tackling major research and translational questions.

**Fig. 3:**
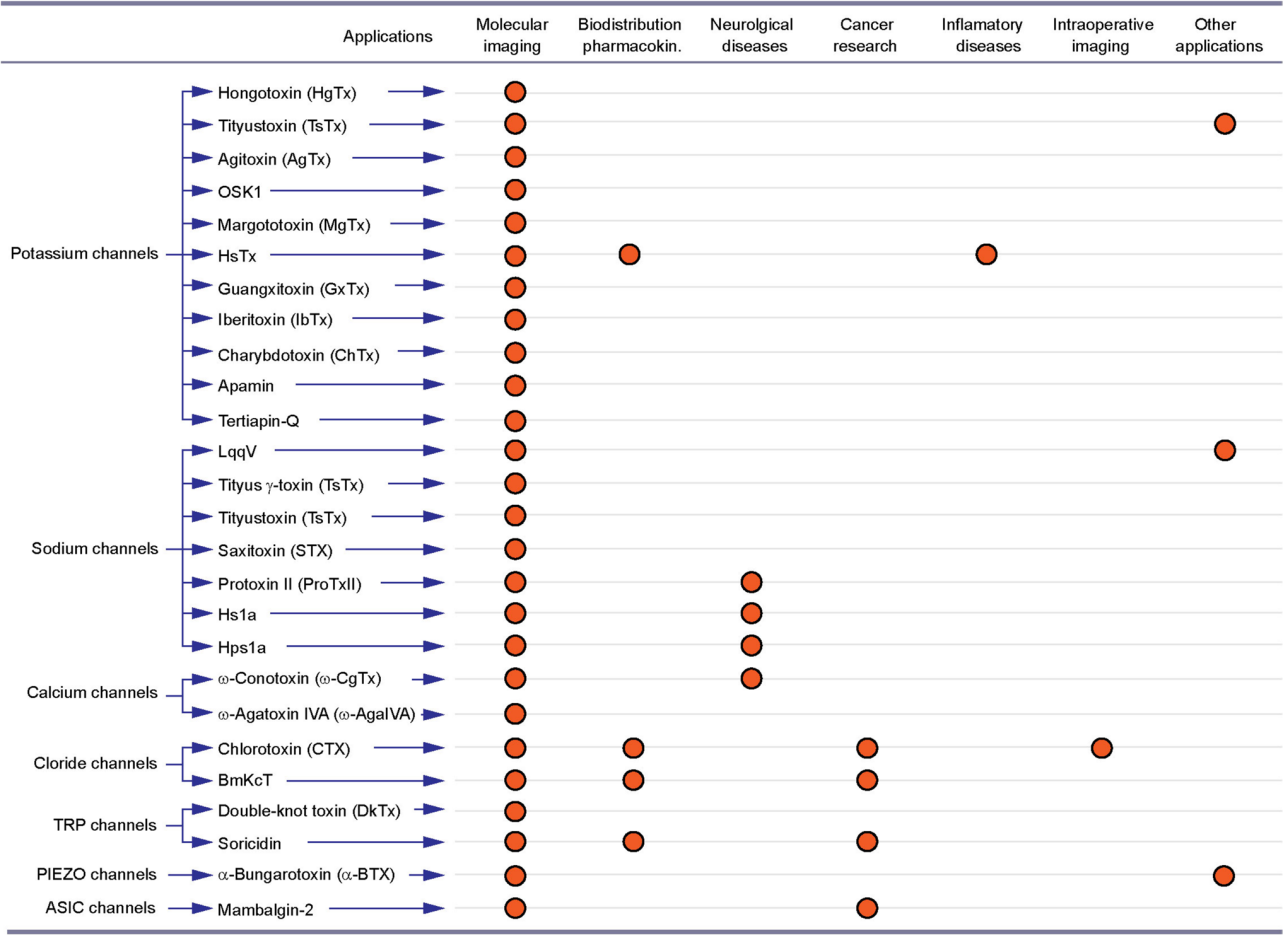
Representation of basic and translational research areas of the use of animal toxin-derived optical probes targeting ion channels. List of ion channels (left column) targeted by labelled animal toxins (middle column) utilised for various research and translational applications (red circle). The details of the applications of each optical probe are described in corresponding sections of the review

Since the first demonstration of the block of action potentials by TTX and inhibition of Na_V_ currents in nerves and muscles [163, 164], biological toxins have played an increasing role in elucidating important facets of the molecular biology and functions of ion channels and receptors, aiding their isolation, cloning and pharmacological characterisation [86, 165–167]. Combined with revolutionary advances in fluorescence probes and nanomaterials and imaging technologies, the expanding portfolio of neurotoxins and their detoxified forms have been recently applied for a wide range of applications for preclinical and clinical imaging (Fig. 3). As reviewed herein, advances in harnessing the potential of neurotoxins for biological imaging have also revealed considerable limitations. These are largely attributed to remarkable potency and harmful effects of toxins, owing to their specific interference with important biological functions of their targets, as well as challenges related to immunological incompatibility and bioavailability. Non-specific interactions with a wide range of other functional proteins, and potential off-site effects, are also of major concern, along with the release of harmful products of fluorophore degradation with phototoxicity. In addition to the limitations associated with biological factors, the widespread use of toxin-based optical probes is also restrained by the physical properties of light and its interactions with the specimen, resulting in attenuation of fluorescence signals, confining high-resolution optical interrogation to the surface of the biological specimen.

 In summary, along with well-recognised ecological, evolutionary and therapeutic dimensions, the ultimate weapons of nature, animal venoms, are becoming of increasing research and diagnostic relevance. Harnessing the full potential of animal toxins and their detoxified forms for biomedical imaging, through systematic research and optimization, is anticipated to improve the efficacy and specificity of toxin-based optical probes utilized in future research.
